# RETRACTED: Huang et al. Adenine Inhibits the Invasive Potential of DLD-1 Human Colorectal Cancer Cell via the AMPK/FAK Axis. *Pharmaceuticals* 2021, *14*, 860

**DOI:** 10.3390/ph19040646

**Published:** 2026-04-21

**Authors:** Chien-Wei Huang, You-Cian Lin, Chia-Hung Hung, Han-Min Chen, Jiun-Tsai Lin, Chau-Jong Wang, Shao-Hsuan Kao

**Affiliations:** 1 Division of Gastroenterology, Department of Internal Medicine, Kaohsiung Armed Forces General Hospital, Kaohsiung 802301, Taiwan; d88442003@gmail.com; 2 Department of Nursing, Tajen University, Pingtung 907101, Taiwan; 3 Surgical Department Cardiovascular Division, China Medical University Hospital, Taichung 404332, Taiwan; 4 School of Medicine, China Medical University, Taichung 404332, Taiwan; 5 Institute of Medicine, College of Medicine, Chung Shan Medical University, Taichung 402306, Taiwan; 6 Institute of Applied Science and Engineering, Catholic Fu Jen University, New Taipei 242048, Taiwan; 7 Energenesis Biomedical Co., Ltd., Taipei 114694, Taiwan; 8 Clinical Laboratory, Chung Shan Medical University Hospital, Taichung 402306, Taiwan

The journal retracts the article, “Adenine Inhibits the Invasive Potential of DLD-1 Human Colorectal Cancer Cell via the AMPK/FAK Axis” [1], cited above.

Following publication, concerns were brought to the attention of the Editorial Office regarding the integrity of figures presented in this publication [1].

In accordance with the journal’s standard procedures, the Editorial Office and Editorial Board conducted an investigation that identified duplication between several panels presented in Figures 2 and 6. Although the authors cooperated with the investigation, the explanations and supporting materials provided were not deemed sufficient by the Editorial Board to resolve the identified concerns. As a consequence, the Editorial Board has lost confidence in the reliability of the reported findings and has decided to retract this publication in accordance with MDPI’s retraction policy.

This retraction was approved by the Editor-in-Chief of the journal *Pharmaceuticals*.

The authors have been informed of this retraction and have indicated their disagreement with this decision.
